# The Role of 8-oxoG Repair Systems in Tumorigenesis and Cancer Therapy

**DOI:** 10.3390/cells11233798

**Published:** 2022-11-27

**Authors:** Chunshuang Li, Yaoyao Xue, Xueqing Ba, Ruoxi Wang

**Affiliations:** 1 Center for Cell Structure and Function, Key Laboratory of Animal Resistance Biology of Shandong Province, College of Life Sciences, Shandong Normal University, Jinan 250014, China; 2 The Key Laboratory of Molecular Epigenetics of Education, School of Life Science, Northeast Normal University, Changchun 130024, China

**Keywords:** oxidative stress, 8-oxoG, tumorigenesis, cancer therapy, synergistic

## Abstract

Tumorigenesis is highly correlated with the accumulation of mutations. The abundant and extensive DNA oxidation product, 8-Oxoguanine (8-oxoG), can cause mutations if it is not repaired by 8-oxoG repair systems. Therefore, the accumulation of 8-oxoG plays an essential role in tumorigenesis. To avoid the accumulation of 8-oxoG in the genome, base excision repair (BER), initiated by 8-oxoguanine DNA glycosylase1 (OGG1), is responsible for the removal of genomic 8-oxoG. It has been proven that 8-oxoG levels are significantly elevated in cancer cells compared with cells of normal tissues, and the induction of DNA damage by some antitumor drugs involves direct or indirect interference with BER, especially through inducing the production and accumulation of reactive oxygen species (ROS), which can lead to tumor cell death. In addition, the absence of the core components of BER can result in embryonic or early post-natal lethality in mice. Therefore, targeting 8-oxoG repair systems with inhibitors is a promising avenue for tumor therapy. In this study, we summarize the impact of 8-oxoG accumulation on tumorigenesis and the current status of cancer therapy approaches exploiting 8-oxoG repair enzyme targeting, as well as possible synergistic lethality strategies involving exogenous ROS-inducing agents.

## 1. Introduction

Most cancer cells possess elevated levels of reactive oxygen species (ROS) due to oncogene activation, abnormal metabolism and mitochondrial dysfunction, among other mechanisms [1,2]. ROS are broadly defined as oxygen-containing chemical species. There are two types of ROS: one is free radicals, including superoxide (O_2_^•−^) and hydroxyl (HO^•^), and the other is nonradical molecules, such as hydrogen peroxide (H_2_O_2_). Mitochondria are considered to be the main site of ROS production, as approximately 2% of the oxygen consumed by mitochondria is reduced to form superoxide [3,4]. A moderate increase of ROS is beneficial to cell proliferation and differentiation [5]; however, excessive accumulation of ROS, known as oxidative stress, can cause oxidative damage to proteins, lipids and DNA [6]. Proteins and lipids are usually degraded and recycled after being oxidized, while oxidized DNA needs to be repaired to ensure the integrity of the genome [6,7]. Damage to DNA, as genetic material, is particularly harmful because it can lead to the destruction of genetic information. Types of oxidative DNA damage include base oxidation, deoxyribose oxidation, apurine/apyrimidine (AP) sites, single-strand breaks (SSBs) and DNA double-strand breaks (DSBs) [8]. Among the four bases, guanine is the most sensitive to ROS because it has the lowest redox potential [9,10]. Accordingly, its oxidation product, 7,8-dihydro-8-oxoguanine (8-oxoG), is the most widespread of the oxidative damage products of all bases and is, thus, taken as a biomarker of oxidative stress. If 8-oxoG is not repaired in time by base excision repair (BER) initiated by 8-oxoguanine DNA glycosylase1 (OGG1), it leads to gene mutation [11]. As we know, tumorigenesis is highly correlated with the accumulation of mutations; therefore, in this study, we intend to systematically discuss the role of 8-oxoG in tumorigenesis.

**Figure 1:**
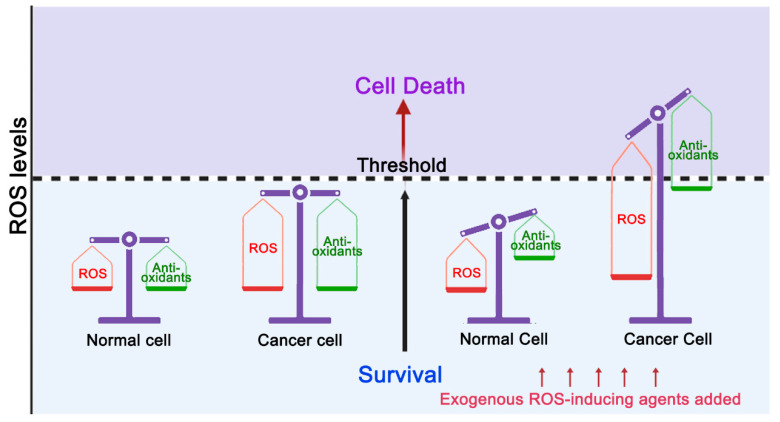
Targeting ROS kills cancer cells. In normal cells, redox homeostasis is accompanied by low levels of ROS. When exogenous ROS-inducing agents are added, normal cells have the ability to increase their antioxidant capacity to avoid ROS levels reaching the cell death threshold. In cancer cells, ROS levels are elevated due to metabolic abnormalities. In order to maintain survival under high ROS pressure, the antioxidant capacity of cancer cells is also accordingly increased. Therefore, cancer cells are more sensitive to ROS, and exogenous ROS-inducing agents may be more likely to cause elevation of ROS above the threshold level, resulting in cancer cell death.

Targeting BER as a tumor treatment strategy is also attracting increasing attention. BER inhibitors can be used both as single agents for tumor therapy or as chemotherapy and radiotherapy sensitizers for synergistic lethality. It is worth noting that in order to escape oxidative damage, tumor cells have also evolved adaptive mechanisms for maintaining redox homeostasis within the death threshold. Therefore, it is believed that disrupting oxidative homeostasis by applying exogenous ROS-inducing agents can lead to massive ROS accumulation and cancer cell death (Figure 1). It has been reported that diverse ROS-inducing agents have an effect on a variety of tumors, although dose-limiting toxic side effects and drug resistance have also been reported [12,13]. For example, β-lapachone (ARQ761 in clinical form), a quinone that promotes ROS production through the futile redox cycle, can cause patients to experience varying degrees of adverse events, such as anemia, fatigue, hypoxia, nausea and vomiting [14]. The futile redox cycle caused by β-lapachone refers to the reduction of β-lapachone by NQO1 and leads to a futile cycling between the quinone and hydroquinone forms, with a concomitant loss of reduced NAD(P)H [15]. Considering the clinical side effects of these drugs, it is necessary to design more efficient treatment strategies, and combination therapy is a very good option. Of note, the 8-oxoG repair system is one of the main repair pathways for ROS-induced oxidative DNA damage. Hence, it seems feasible to target any step of the 8-oxoG repair pathway and to amplify the death signal through the combined use of 8-oxoG repair enzyme inhibitors with ROS-inducing agents to improve tumor therapy efficacy. Therefore, in this review we summarize the current use of targeted 8-oxoG repair enzyme inhibitors in tumor therapy and discuss therapeutic strategies that target 8-oxoG repair enzyme inhibitors in combination with ROS-inducing agents.

## 2. Systems for Formation and Elimination of 8-oxoG

**Figure 2:**
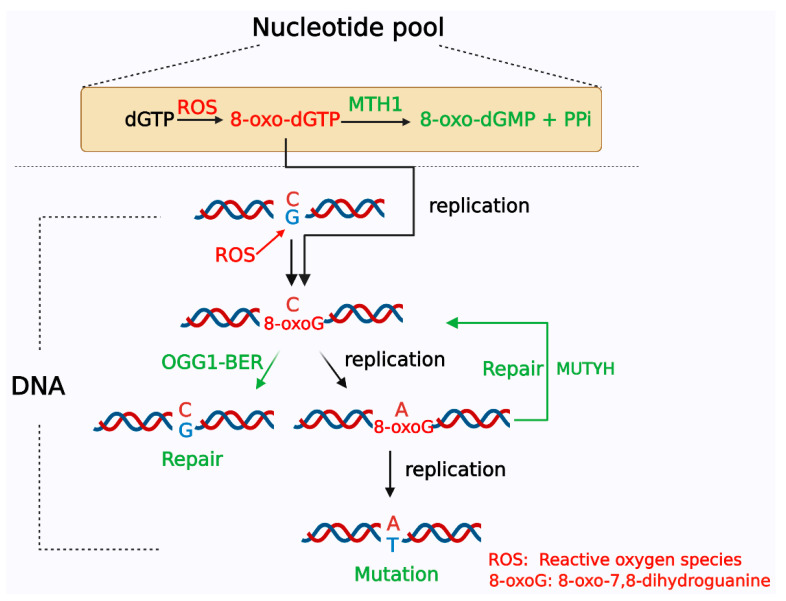
The repair pathway of 8-oxoG. ① MTH1 is responsible for the hydrolysis of 8-oxo-dGTP in the nucleotide pool to prevent the incorporation of 8-oxo-dGTP into DNA through replication. ② Guanine in DNA can be directly oxidized to form 8-oxoG. 8-Oxoguanine DNA glycosylase1 (OGG1)-initiated base excision repair (BER) is responsible for removing 8-oxoG from DNA. If adenine is inserted on the opposite side of 8-oxoG during replication, it is removed by the MutY homolog (MUTYH). If cytosine is inserted opposite 8-oxoG after adenine is cleaved, OGG1 initiates BER to repair 8-oxoG, and if adenine is inserted opposite 8-oxoG, the BER initiated by MUTYH continues again.

Under ROS attack, guanines in DNA or free 2′-deoxyguanosine5′-triphosphate (dGTP) in the nucleotide pool undergo oxygen addition at the 8th carbon to become 8-oxoG or 8-oxo-dGTP, respectively [16,17]. Compared with guanine in DNA, free dGTP in the nucleotide pool is more susceptible to oxidation. Previous research showed that free dNTPs in the nucleotide pool are up to 13,000-fold more susceptible to oxidative damage than bases in duplex DNA [18]. During DNA replication, unrepaired 8-oxoG can pair with either adenine or cytosine, resulting in G:C to T:A transversion [7,19]. Meanwhile, DNA polymerase can also insert 8-oxo-dGTP into DNA opposite adenine and cytosine, which can also introduce mutations and cause the accumulation of 8-oxoG in DNA [19,20,21]. To avoid the incorporation of oxidized bases into DNA, 8-oxo-dGTP, in the nucleotide pool of eukaryotic cells, can be hydrolyzed to 8-oxo-dGMP and pyrophosphate by MTH1, also known as nudix hydrolase 1 (NUDT1), which is a homolog of the MutT protein in *E. coli* [22,23] (Figure 2). *MutT* gene deficiency increases the occurrence of A:T to C:G transversion mutations 1000-fold [24]. Once MTH1 binds to the substrate 8-oxo-dGTP, the hydrolysis reaction occurs immediately to generate 8-oxo-dGMP; therefore, Stenmarks’ group analyzed the complex structure of MTH1 and 8-oxo-dGMP and found that 8-oxo-dGMP was bound in the active site pocket composed of residues Leu9, Phe72, Met81, Val83, Trp117, Phe27, Trp123 and Phe139. Moreover, Asn33, Asp119 and Asp120 were the key amino acid residues in the interaction between the target protein and the substrate [25]. Nissink et al. further confirmed that Asp119, Asp120, Asn33, Met81 and Phe27 are key amino acid residues in MTH1 for recognizing and binding substrates, especially Asp119 and Asp120 [26]. These data provided the structural basis for MTH1 inhibitor design. The 8-oxoG formed in DNA is mainly repaired through the BER pathway by OGG1, which is a functional protein homolog of *E. coli* MutM/Fpg [27,28,29]. If OGG1 fails to repair 8-oxoG in DNA and adenine is inserted on the opposite side of 8-oxoG during replication, the MutY homolog (MUTYH) removes the adenine. If cytosine is inserted opposite 8-oxoG after adenine is cleaved, OGG1 initiates BER to repair 8-oxoG, and if adenine is inserted opposite 8-oxoG, the BER initiated by MUTYH continues (Figure 2) [30,31,32].

**Figure 3:**
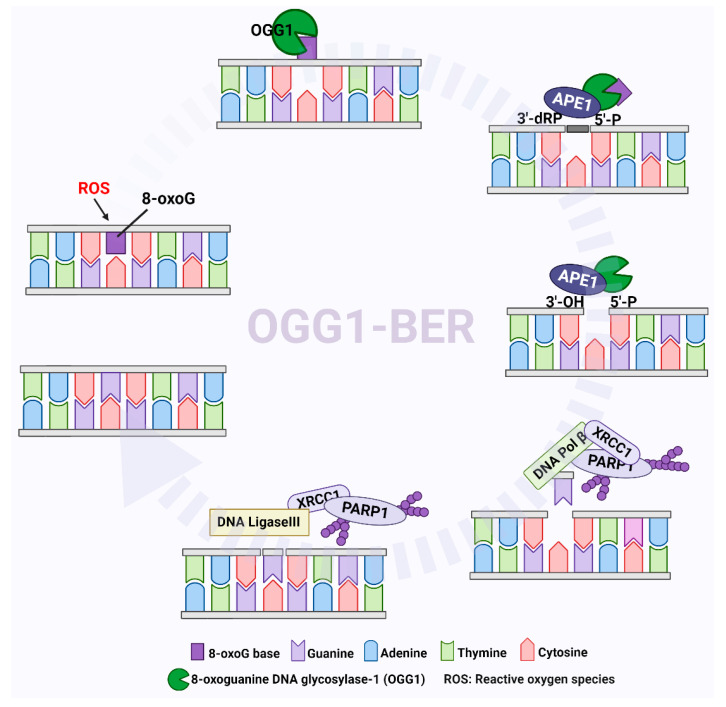
OGG1-initiated DNA base excision repair pathway. OGG1 recognizes 8-oxoG and cleaves N-glycosidic bonds to excise 8-oxoG, generating an apurinic/apyrimidinic (AP)-site(s). Apurinic/apyrimidinic endonuclease 1 (APE1) processes the single-strand gap through its phosphodiesterase activity to form the 3′-OH terminus. PARP-1 recognizes the single-strand gap as a single-strand break, catalyzing ADP-ribose units to PARP1 itself, forming charged poly(ADP-ribose) (PAR) chains. PAR chains recruit X-ray repair cross-complementing 1 (XRCC1) with DNA polymerase β (POL β) and DNA ligase III (LIG3) to sites of DNA single-strand breaks (SSBs) to complete the repair process.

The OGG1-initiated BER (OGG1–BER) pathway is a multi-step event involving the recognition of oxidized bases, changes in DNA structure, the introduction of damaged bases into OGG1 and the base-binding region, the excision of bases and strand cleavage [33,34]. OGG1 is a bifunctional DNA glycosidase that recognizes 8-oxoG and exerts glycosidase activity to cleave N-glycosidic bonds to excise 8-oxoG, resulting in the formation of AP sites. After 8-oxoG is removed, OGG1 cleaves the DNA phosphate backbone at AP sites through its AP-lyase activity, forming 3’-phospho-α,β-unsaturated aldehyde (3’dRP) and 5’-phosphate termini. Since the 3’dRP terminus cannot act as a primer for DNA polymerase, apurinic/apyrimidinic endonuclease 1 (APE1) must exert its phosphodiesterase activity to form the 3’-OH terminus, creating a nucleotide gap equivalent to a single-strand DNA nick. Subsequently, DNA polymerases, particularly DNA polymerase β (POL β), then insert guanine at the gapped site via a polymerization reaction, and the resulting nicks are sealed by DNA ligase to complete the repair process (Figure 3) [35,36,37].

It was reported that poly(ADP-ribose) polymerase 1 (PARP1) is also involved in the BER process. PARP1 can recognize DNA nicks produced by APE1 cleavage as DNA single-strand breaks and then use NAD+ as substrates to catalyze ADP-ribose units to PARP1 itself, forming charged poly(ADP-ribose) (PAR) chains [38]. PAR chains further recruit X-ray repair cross-complementing 1 (XRCC1) [39,40] with POL β and DNA ligase III (LIG3) to the SSBs to complete the repair process (Figure 3) [41,42,43]. In addition to recruiting downstream factors, studies also showed that SSBs can be protected from nuclease degradation through combination with PARP1 [44,45]. It is worth mentioning that XRCC1 is a key factor in the BER process. Although XRCC1 has no enzymatic function of its own, it can, through its BRCT domains, interact with other repair proteins involved in BER, such as DNA ligase IIIα, Polβ and PARP1 [46]. Moreover, XRCC1 depletion significantly increases β-lapachone-induced DNA double-strand breaks and dramatically sensitizes cells to β-lapachone [47,48]. This suggests that inhibiting the recruitment of XRCC1 to DNA lesions, or disrupting the interaction of XRCC1 with other repair enzymes, may be a feasible strategy for tumor therapy. At present, the role of PARP1 in BER is still controversial. For example, Strom et al. showed that SSBs induced by alkylating agent dimethyl sulfate (DMS) rapidly accumulated in PARP inhibitor-treated cells. However, the same result was not observed in siRNA-treated PARP1-cells. This seems to indicate that there is no immediate role for PARP1 in BER, but that PARP inhibitors trap PARP on the SSB intermediate formed during BER [49]. Reynolds et al. demonstrated that the involvement of PARP1 in BER is dependent on the type of lesion, and the repair of SSBs and purine base damage occurs through a sub-pathway of BER that requires both XRCC1 and PARP1. However, the repair of pyrimidine base damage may require XRCC1, but not PARP1 activity [40].

As we know, besides the nucleus, mitochondria also contain DNA. Mitochondrial DNA (mtDNA) is more inclined to be oxidized because it is close to the electron transport chain, compared with nuclear DNA. It was shown that the level of oxidized bases in mtDNA is 2–3 times higher than that in the nucleus [50,51]. Particularly, in mtDNA high levels of 8-oxoG have been detected [52,53], up to 16 times higher than in nuclear DNA [54]. The repair of 8-oxoG in mitochondria also mainly depends on OGG1, MTH1 and MUTYH [55]. In the BER process in mitochondria, after OGG1 recognizes and cleaves 8-oxoG, APE1, DNA polymerase γ (Pol-γ) and DNA ligase III participate in the subsequent repair process and these BER proteins are encoded by nuclear genes and then transported to mitochondria [55,56,57]. It is worth mentioning that there are two isoforms of OGG1 proteins in mitochondria, namely α-OGG1 and β-OGG1. The OGG1 that we mentioned in the full text is α-OGG1 (which also continues to be written as ‘OGG1’), which has both nuclear localization signal and mitochondrial localization signal [58]. In addition to exerting DNA glycosidase activity in the nucleus, it can also be imported into mitochondria to exert DNA glycosidase activity [55]. The β-OGG1 is only located in mitochondria and since β-OGG1 lacks the C-terminal αO helix present in α-OGG1, β-OGG1 is not responsible for 8-oxoG incision in mitochondria. The content of β-OGG1 was found to be significantly higher than that of α-OGG1 in mitochondria [54,59]. The function of β-OGG1 has been constantly explored.

## 3. The Effect of Accumulation of 8-oxoG on Tumorigenesis

It was proposed that tumorigenesis is highly correlated with the accumulation of mutations and their extent [60,61]. Additionally, the persistence of 8-oxoG in the genome may increase the risk of spontaneous mutagenesis, resulting in the malignant transformation of cells [62,63]. Numerous studies have shown that a deficiency of BER enzymes increases the probability of tumorigenesis. For instance, compared with wild-type mice, *Mth1*-deficient mice developed a greater number of tumors in their lungs, livers and stomachs 18 months post-birth, and an analysis of the total number of tumor-bearing mice showed that the percentage of *Mth1*-deficient mice (36%) was significantly higher than that of wild-type mice (11%) [64]. The quantity of 8-oxoG in DNA and G:C→T:A transversion mutations were significantly higher in *Ogg1* knockout mice than in wild-type mice, and in the 18 months following birth, the incidence of spontaneous lung adenoma/carcinoma was significantly elevated in *Ogg1* knockout mice, and the percentage of tumor-bearing *Ogg1* knockout mice was also 5 times higher than that observed in wild-type mice [65]. In addition, administering the oxidative agent potassium bromate (KBrO3) to *Ogg1*^+/+^ and *Ogg1*^−/−^ mice, via drinking water, resulted in greatly increased abundance of 8-oxoG, which mainly caused G:C→T:A transversion in the kidney DNA of *Ogg1*^−/−^ mice [66]. Moreover, compared with wild-type mice, *Ogg1* knockout mice also exhibited an increased occurrence of skin carcinogenesis accompanied by a significant increase in 8-oxoG under UVB irradiation [67]. Consistent with this, Kakehashi et al. employed a multi-organ carcinogenicity bioassay to demonstrate that *Ogg1* knockout mice had increased susceptibility to multi-organ carcinogenesis [68].

Besides OGG1 and MTH1, MUTYH is also considered to play an important role in preventing G:C to T:A transversion by cleaving the adenine opposite 8-oxoG in mammalian cells [69,70]. Previous studies have shown that the incidence of intestinal tumors is significantly higher in *Mutyh*-null mice than in wild-type mice, whether under conditions of spontaneous or chronic oxidative stress [71]. It was shown that deficiencies in *MUTYH* and *OGG1* predisposed 65.7% of mice to tumors, primarily lung and ovarian tumors and lymphomas, and G to T mutations were observed in the *K-ras* oncogene in 75% of lung tumors, whereas none were found in the adjacent normal tissues [72]. *Mth1*/*Ogg1*/*Mutyh* triple knockout (TOY-KO) mice were also established and found to have a significantly shortened lifespan, with more than 35% carrying macroscopically distinguishable tumors [73]. These data strongly suggest that the accumulation of 8-oxoG, which promotes G:C to T:A transversion in the genome, can lead to tumorigenesis, and these findings may be beneficial in developing strategies for tumor prevention.

Due to the critical role of OGG1 in 8-oxoG repair, the impact of *OGG1* gene mutations, such as polymorphisms, have also received special attention, especially in the context of tumor development. It was reported that a single-nucleotide polymorphism at position 1245 in exon 7 of the *OGG1* gene resulted in a serine to cysteine amino acid substitution at position 326 of the protein (C > G, Ser326Cys) [74]. The evidence suggested that serine-to-cysteine changes increased genome instability and reduced 8-oxoG repair efficiency, possibly due to the formation of a disulfide bond in Cys326 oxidation and the reduced affinity between Cys326 OGG1 and 8-oxoG [74,75,76]. In addition, Janik et al. reported that 8-oxodG levels in patients with the Cys/Cys genotype were higher than in those with the Ser/Ser genotype, both in lung tissue DNA (in normal lung and tumor cells) and in leukocyte DNA [77]. These data seem to strongly suggest that OGG1 S326C may contribute to the accumulation of genetic mutations and the development of cancer. Many studies have explored the link between OGG1 Ser326Cys polymorphism and cancer susceptibility; however, the results are controversial. For instance, Wei et al. [78] reported that OGG1 Ser326Cys polymorphism was significantly associated with the risk of lung cancer. However, Duan et al. [79] performed a meta-analysis with more studies, and no obvious association between OGG1 Ser326Cys polymorphism and lung cancer risk was found. In addition, Wei et al. [78] showed that OGG1 Ser326Cys polymorphism was not related to colorectal cancer, but another study [80] showed that the two were related. Furthermore, OGG1 Ser326Cys seems to play a role in specific cancer types and a specific population. For example, the recent findings of Zhou et al. [81] suggested that OGG1 Ser326Cys polymorphism may be a risk factor for lung, digestive system and head and neck cancers; nevertheless, no association was observed in breast, prostate and bladder cancers, and the association between OGG1 Ser326Cys polymorphism and the susceptibility of digestive system cancers was found to only exist in Asian populations. Taken together, more samples and further studies are needed to elucidate the relationship between OGG1 Ser326Cys polymorphism and tumor susceptibility.

Notably, there is a close link between inflammation reactions and digestive system cancers, and beyond digestive system cancers, inflammatory responses appear to play a nonnegligible role in every stage of tumor development, including tumor initiation, progression, malignant proliferation, invasion of surrounding tissues and distant metastasis. Both inflammation and tumor development are accompanied by oxidative stress. Coincidentally, another widely recognized and studied function of OGG1 is in promoting the expression of inflammatory genes [82]. Moreover, although *Ogg1* knockout mice had higher 8-oxoG levels than wild-type mice, their mitochondrial respiration rate and ROS generation were not significantly altered, and although *Ogg1* knockout mice had no obvious pathological phenotype, they unexpectedly showed a reduced degree of inflammatory response. In addition, *Ogg1* knockout mice exhibited reduced serum IgG2a levels in response to bacterial infections, accompanied by reductions in the expression of chemokine Mip-1α the Th1 cytokines interleukin-12 (IL-12) and tumor necrosis factor-α (TNFα) [83,84]. Similarly, compared with wild-type mice, the lungs of *Ogg1* knockout mice stimulated by ovalbumin showed reduced inflammatory cell infiltration capacity and oxidative stress levels [85]. These data suggest that the pro-inflammatory role of OGG1 could be an etiological explanation linking 8-oxoG to tumor susceptibility. More specific experiments are needed to explore this possibility.

It was shown that the loss of OGG1 can also cause the accumulation of 8-oxoG in mitochondria and a large number of studies showed that the accumulation of mtDNA damage and mutation, leading to mitochondrial dysfunction, might be related to aging, age-associated degenerative diseases, such as Parkinson’s disease and tumorigenesis [52,86,87,88]. For example, using *Ogg1*^−/−^ mice, researchers found that liver mtDNA from these animals accumulated 20 times more 8-oxoG than wild-type mice [89]. In addition, Leon et al. found that MTH1/OGG1 deficiency significantly increased the accumulation of 8-oxoG in mtDNA of cortical neurons cultured in the absence of antioxidants, which caused mitochondrial dysfunction and impaired neuritogenesis in cultured adult mouse cortical neurons [90]. Kim et al. showed that, compared to wild-type, mice over-expressing mtOGG1 transgene (*mtOgg1^tg^*) had diminished asbestos- and bleomycin-induced pulmonary fibrosis that was accompanied by reduced lung and alveolar epithelial cell mtDNA damage and apoptosis [91]. Yuzefovych et al. used mice lacking OGG1 (KO), mice overexpressing human OGG1 subunit 1α in mitochondria (Tg), and mice simultaneously lacking OGG1 and overexpressing human OGG1 subunit 1α in mitochondria (KO/Tg) to test, and found that Tg and KO/Tg mice developed significantly smaller tumors than KO and wildtype mice after 16 weeks [88]. Furthermore, lungs from Tg mice exhibited nearly a 15-fold decrease in the average number of metastatic foci compared to wild-type mice and primary tumors isolated from Tg mice had reduced total and mitochondrial oxidative stress, diminished mtDNA damage, and increased mitochondrial function [88]. In conclusion, the accumulation of 8-oxoG and the activity of OGG1-BER in mitochondria also closely affect the stability of mtDNA, the function of mitochondria and even the occurrence and development of tumors. 

## 4. Targeting 8-oxoG Repair Systems as a Tumor Therapy Strategy

**Figure 4:**
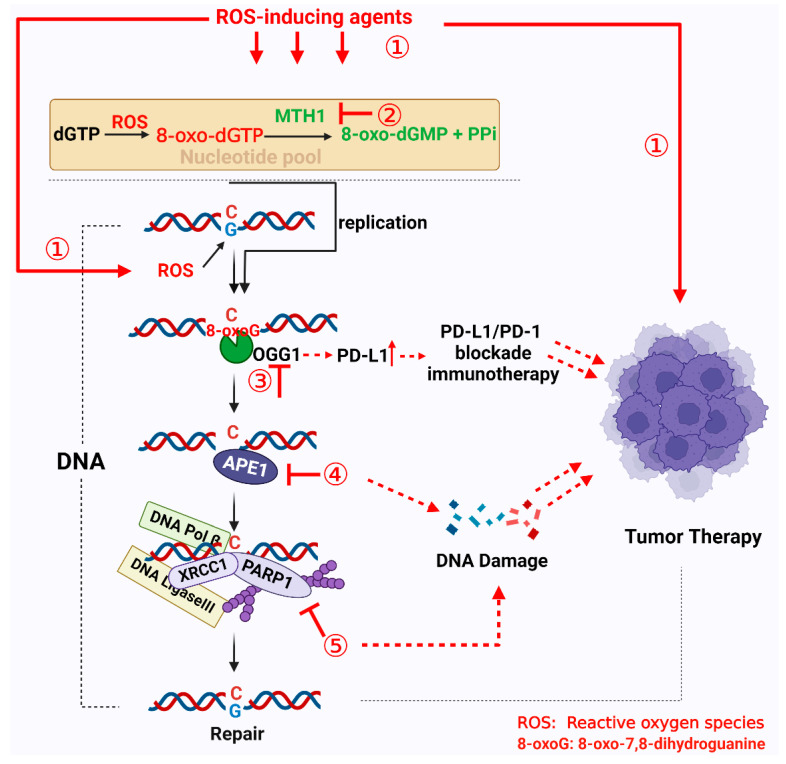
Potential therapeutic strategies for cancer targeting 8-oxoG repair systems. ① ROS-inducing agents cause tumor cell death by increasing intracellular ROS levels above the death threshold. ② Inhibition of MTH1 activity increases levels of 8-oxoG incorporation in the genome, which, in turn, increases DNA fragmentation leading to cell death. ③ Inhibiting the BER activity of OGG1. Under oxidative stress conditions, OGG1 significantly augments PD-L1 upregulation. OGG1 inhibitors may potentiate the therapeutic effect of PD-L1/PD-1. ④ Inhibition of APE1 resulting in the accumulation of AP sites, can hinder DNA replication and potentially cause cytotoxic DNA damage. ⑤ Inhibition of PARP1 activity leads to the accumulation of unrepaired SSBs and their conversion into DSBs, through replication fork collision.

Targeting BER enzymes with inhibitors, either as monotherapy agents or in combination with radiotherapy and/or chemotherapy, is a currently employed strategy for treating tumors [92]. Additionally, as mentioned above, the use of exogenous ROS-inducing agents to induce ROS accumulation in tumor cells, thus leading to cell death, has become a promising tumor therapy strategy. Moreover, OGG1–BER plays an important role in this process. Consequently, in this section, we summarize the current use of targeted 8-oxoG repair enzyme inhibitors in tumor therapy and discuss therapeutic strategies for targeting 8-oxoG repair enzyme inhibitors in combination with ROS-inducing agents (Figure 4).

### 4.1. Targeting MTH1

To maintain genome stability, one of the main means by which tumor cells resist high levels of oxidative stress is by increasing the expression of MTH1, which can effectively hydrolyze 8-oxo-dGTP in the nucleotide pool to protect cells. Studies have shown that MTH1 is highly expressed in a variety of tumor cells, such as those of lung, colon, breast and pancreatic cancers [93,94,95,96]. In addition, Helleday’s group used siRNA to knock down MTH1 in human osteosarcoma U2OS cells and normal VH10 cells, and then detected levels of 8-oxoG and the survival of cells. Their results showed that MTH1 depletion in cancer cells resulted in the accumulation of 8-oxoG in DNA, and the addition of OGG1 increased DNA strand breaks, while temporarily inhibiting the survival and viability of tumor cells. It is worth noting that the same results were not observed in VH10 cells [97]. Thus, MTH1 is highly expressed and extremely essential for tumor tissue cells, but not normal tissue cells [97,98]. Moreover, compared with wild-type mice, the lifespan of *Mth1* knockout mice was not significantly changed [64]. This further supports the view that normal cells do not depend on MTH1 for survival, unlike tumor cells, which are extremely dependent on MTH1. Notably, our previous results suggest that, under oxidative stress, 8-oxoG repair by OGG1 can exacerbate cell death through the parthanatos cell death pathway [99]. These features provide a therapeutic strategy against tumors that involves the inhibition of MTH1 function to result in cytotoxicity caused by the accumulation of 8-oxoG in DNA.

A variety of MTH inhibitors have been developed that can specifically bind to the active sites of MTH1 and inhibit its activity; these include the TH series of TH588, TH287 and TH1579 (Karonudib in clinical form), (S)-crizotinib and IACS-4619 and IACS-4759 [97,98,100,101] (Table 1). Numerous studies have shown that MTH1 inhibitors have positive effects on many types of tumors, such as osteosarcoma [102], hepatocellular carcinoma [103], B-cell lymphoma [104] and gastric cancer [105]. However, the identity of the specific targets of MTH1 inhibitors remains controversial. For instance, it was shown that silencing MTH1 did not affect the survival of melanoma cells, and TH588 killed melanoma independent of MTH1 inhibition [106]. In addition, a study by Kettle’s group revealed that MTH1 was dispensable for cancer cell survival, and their synthesized MTH1 inhibitor killed cancer cells in an MTH1-independent manner [107]. These seemingly paradoxical results suggest that the specific mechanism by which MTH1 inhibitors target MTH1 in tumor therapy, and the mechanism by which MTH1 inhibitors induce cell death, still need to be explored.

Additionally, it is also worth noting that the presence of MTH1 protects the brain and prevents the occurrence of neurodegenerative diseases [90,108,109]. For example, Ventura et al. found that MTH1 expression protected mitochondria from a Huntington’s disease-like impairment [110]. Likewise, Yamaguchi et al. treated *Mth1*^−/−^ mice with 1-methyl-4-phenyl-1,2,3,6-tetrahydropyrine (MPTP) and found that MTH1 protected the mitochondrial DNA of striatal nerve terminals of dopamine neurons, which are associated with Parkinson’s disease [111]. Since the presence of MTH1 protects the brain, long-term inhibition may lead to neurodegenerative diseases associated with oxidative stress, so it is important to consider how to reduce this possible disadvantage of using MTH1 inhibitors in cancer therapy. Improving the targeting of MTH1 inhibitor following delivery, and pursuing synergistic lethality through combination with other antitumor drugs, may be useful options.

As mentioned above, 8-oxoG is the most widespread DNA oxidation product, due to the lower redox potential of guanine. Moreover, it has been shown that some ROS-inducing agents can cause the accumulation of 8-oxoG in tumor cells. For instance, Wang et al. found that treatment with alantolactone, a natural compound that inhibits thioredoxin reductase to induce ROS accumulation, could lead to increased 8-oxoG in tumor cells, and the inhibition of OGG1 by the knockdown, or addition, of an OGG1 inhibitor and significantly improve cell viability [112]. Consistently, Chakrabarti et al. also found that OGG1 depletion rescued cells from lethality induced by another ROS-inducing agent, β-lapachone [47]. These results indicate that the repair of 8-oxoG by OGG1 plays a significantly critical role in oxidative stress-induced cell death. Considering the function of MTH1 and its high expression in tumor cells, together with the role of 8-oxoG in ROS-mediated tumor therapy, it may seem highly likely that the selectivity and efficiency of tumor therapy can be improved through combined administering of ROS-inducing agents with MTH1 inhibitors. Photodynamic therapy (PDT) is a clinically approved oncologic intervention approach that causes an increase in ROS levels. It was reported that the MTH1 inhibitor TH588 can significantly promote photodynamic-induced cellular apoptosis by increasing 8-oxoG in nuclei and mitochondria [113]. In addition, Centio et al. used the inv(16)/KITD816Y AML mouse model, mimicking the genetics of acute myelocytic leukemia (AML) patients exhibiting a poor response to standard chemotherapy, to explore the effects of a combination of MTH1 inhibitor TH1579 with ROS-inducing chemotherapy. The results showed that the combinatorial treatment of inv(16)/KITD816Y AML cells with the MTH1 inhibitor TH1579 and ROS-inducing agents could significantly increase DNA damage through the incorporation of oxidized nucleotides into DNA. Moreover, TH1579 and chemotherapy synergistically inhibited the growth of clonogenic inv(16)/KITD816Y AML cells without substantially inhibiting normal clonogenicity bone marrow cells [114]. These results suggest that the combination of MTH1 inhibitors with ROS-inducing agents represents a potent tumor therapeutic window, and more preclinical experiments need to be carried out to support this notion.

### 4.2. Targeting OGG1

As the main 8-oxoG repair enzyme, OGG1 inhibitors have also been widely studied; however, at present, treatment with OGG1 inhibitors has not produced the outstanding effects in tumor treatment seen with MTH1 inhibitors, although there were obvious effects in inhibiting inflammatory responses [82,115]. As mentioned above, OGG1 depletion, or OGG1 inhibition, can rescue cells from lethality resulting from ROS-inducing agents; therefore, the role of OGG1 inhibitors in tumor therapy still needs to be further elucidated. Interestingly, similarly to how OGG1 affects inflammatory gene expression, OGG1 also affects the expression of programmed death-ligand 1 (PD-L1) in cancer cells.

PD-L1 is the ligand of programmed cell death-1 (PD-1), which is expressed in activated T cells as well as B cells [116]. The binding of PD-L1 with PD-1 inactivates T cells, thus tumor cells avoid the killing effect of T cells, leading to tumor immune escape [117,118]. Therefore, use of monoclonal antibodies that target PD-1 or PD-L1 to disrupt their interaction are an effective strategy in tumor therapy [119]. Monoclonal antibodies currently show promising effects in different tumors [120,121]. However, in clinical practice, only a limited number of patients benefit from a long-term response, while others do not respond well, or eventually develop resistance [122]. Therefore, in order to improve tumor treatment efficiency, it is important to understand the mechanism of PD-L1 expression and to develop therapeutic strategies.

Recently, Shibata’s team found that H_2_O_2_ could upregulate PD-L1 expression in cancer cells and prompt the depletion of BER enzymes, particularly DNA glycosylases, such as NTH1, OGG1 and NEIL1, which significantly augments PD-L1 upregulation in response to H_2_O_2_ by activating ATR/Chk1 signaling at the DNA replication fork collapse, resulting in cytotoxic DNA damage [123]. In addition, an analysis of The Cancer Genome Atlas (TCGA) showed that the expression of most BER/SSBR genes exhibits a negative correlation with PD-L1 expression [123]. This suggests that oxidative DNA damage is involved in the upregulation of PD-L1 in response to exogenous oxidative stress, and combination with an OGG1 inhibitor may improve tumor therapy strategies targeting PD-L1/PD-1. Additionally, it was shown that ROS-modulating drugs are involved in the expression of PD-L1 [122,124]. For instance, arsenic trioxide increased PD-L1 expression in a dose-dependent manner in IL-60 cells [125]. Furthermore, β-lapachone treatment enhanced tumor immunogenicity and increased T cell infiltration and tumor-specific T cell responses when combined with PD-L1 blockade [126]. This indicates that ROS-modulating drugs could potentially overcome therapeutic PD-L1/PD-1 blockade resistance. As such, using a combination of ROS-inducing agents and OGG1 inhibitors to promote PD-L1 expression may be an attractive strategy to overcome therapeutic PD-L1/PD-1 blockade resistance and may, therefore, have significant therapeutic implications.

### 4.3. Targeting APE1

APE1 is a key enzyme responsible for cleaving AP sites to generate an SSB during BER. APE1 has not only AP lyase activity but also transcriptional regulatory activity involving the redox-mediated modulation of transcription factors [127]. APE1 is a good target for blocking BER because it is responsible for the cleavage of more than 95% of AP sites in cells [128]. Deletion or functional inhibition of APE1 results in the accumulation of AP sites, which can hinder DNA replication and potentially cause cytotoxic DNA damage [129]. Additionally, the disruption of APE1 function increases cellular susceptibility to alkylators and antimetabolites [130], and Wang et al. found that APE1 depletion significantly enhanced the sensitivity of A549 cells to cisplatin, due to increased cell apoptosis [131]. Based on this, a variety of APE1 inhibitors were developed for cancer therapy, such as methoxyamine (MX, TRC102 in clinical form), CRT0044876 compound, AR03 and gossypol [132] (Table 1). Methoxyamine does not directly act on APE1 but interacts with aldehydes at AP sites, making them refractory to APE1 binding and causing the accumulation of AP sites [133]. Results using a preclinical model showed that methoxamine can enhance the antitumor activity of chemotherapeutic drugs, such as temozolomide, pemetrexed and fludarabine, which can generate AP sites [134,135,136]. Additionally, phase I clinical results for pemetrexed + methoxyamine [135] and temozolomide + methoxyamine [137] showed that these combinations were safe and adequately tolerated. Besides methoxyamine, another APE1 inhibitor, CRT0044876 compound (7-nitroindole-2-carboxylic acid), specifically inhibits APE1 activity by binding to the active site of APE1, and CRT0044876 can potentiate the cytotoxicity of several DNA base-targeting compounds by promoting the accumulation of unrepaired AP sites [138]. These results suggest that, in targeting APE1 or AP site inhibition for tumor therapy, DNA glycosidase is required for initiating BER and creating AP sites. In this case, the use of MTH1 inhibitors or ROS-inducing agents in combination with APE1/AP site inhibitors may be an attractive new approach to improve therapeutic outcomes.

### 4.4. Targeting PARP1

Although there is debate as to whether PARP1 is involved in the BER pathway, PARP1 inhibitors are significantly effective in tumor therapy. There is evidence that BRCA1- and BRCA2-deficient cells, which effectively perform homologous recombination (HR), are acutely sensitive to PARP inhibitors [139,140]. However, the anticancer mechanism of PARP1 inhibitors is not yet fully understood. It was shown that in HR-deficient cancers, including BRCA1- or BRCA2-deficient cancers, the inhibition of PARP1 could lead to the accumulation of unrepaired SSBs and their conversion into DSBs through replication fork collision [141]. Another possible mechanism is that PARP1 can lead to the trapping of DNA–PARP-1 complexes. PARP1 inhibitors cause a conformational change in PARP1/2, leading to the stabilization of the reversible association of PARP-1/2 with DNA, which is considered to be the trapping of DNA–PARP1 complexes. Once DNA–PARP complexes become trapped at SSBs, they are converted into lethal DSBs in HR-deficient cells, via replication fork collision, leading to cell death [142].

At present, there are many kinds of PARP inhibitors, such as olaparib, talazoparib, niraparib, rucaparib and veliparib (Table 1), which have shown excellent therapeutic effects in clinical trials. Moreover, olaparib was approved by the FDA for the treatment of BRCA-deficient tumors. With the exception of monotherapy agents for BRCA-mutated cancers, PARP1 inhibitors can also be used as collaborators with radiotherapy/chemosensitizers, such as paclitaxel, bevacizumab and topoisomerase inhibitors in combination therapy for various cancer types. Interestingly, it was shown that the deletion of OGG1 or MUTYH significantly attenuated the cytotoxicity of PARP1 inhibitor olaparib against BRCA1-depleted or BRCA1-mutated cells [143]. This suggests that 8-oxoG repair enzymes contribute to the function of PARP1 inhibitors for tumor therapy. As such, utilizing PARP1 inhibitors to block BER induced by ROS-inducing agents, to enhance replication stress and DSB accumulation leading to apoptosis, may be an attractive therapeutic strategy. A recent study by Zou’s group showed that administering of nontoxic doses of alantolactone (ATL), a natural compound inducing ROS accumulation, together with the PARP inhibitor olaparib, led to markedly synergized effects in terms of synthetic lethality; this phenomenon was not observed in noncancer cell lines [112]. Additionally, treatment with the ROS scavenger NAC and OGG1 knockdown significantly reduced synergistic cytotoxicity, indicating that a significant portion of synergistic cytotoxicity resulted from the repair of 8-oxoG by OGG1 [112]. Likewise, several studies suggest that PARP1 inhibitors, in combination with ROS-inducing agents, can result in synthetic lethality, accompanied by the accumulation of DNA damage, such as phenethyl isothiocyanate, alkannin and β-lapachone [144,145,146]. Both PARP1 inhibitors and ROS-inducing agents suffer from challenges due to drug resistance, and this combination may be a good option to improve therapeutic efficacy and reduce dose-limiting toxicities.

As mentioned above, the repair of 8-oxoG in mitochondria is also related to the OGG1-BER system. Thus, inhibitors of 8-oxoG repair enzymes may also cause mtDNA damage, leading to mitochondrial dysfunction and affecting the survival and development of tumor cells. This may be a strategy for tumor therapy targeting mtDNA damage. Of course, the role of mitochondrial 8-oxoG-OGG1-BER in tumor therapy remains to be further clarified and explored.

## 5. Conclusions and Future Prospects

There are many factors involved in the development of cancer; spontaneous mutations resulting from the accumulation of 8-oxoG have been observed to promote carcinogenesis. This explains tumorigenesis from the perspective of oxidative DNA damage-induced gene mutation; however, the role of 8-oxoG in tumorigenesis and development still needs to be explored. For example, concerning the presence of 8-oxoG in the genome, it is unclear whether or not it is bound by OGG1 to promote the expression of inflammatory genes and induce tumorigenesis via an inflammatory environment. Currently, relatively few 8-oxoG-BER inhibitors have been entered in clinical trials, and there are some problems that need to be addressed. For example, BER is also necessary for normal tissues, so it is essential to consider designing a therapeutic regimen to avoid the toxic side effects caused by BER inhibitors. The most widespread product of DNA oxidation is 8-oxoG, so the high oxidation levels in tumor cells provide an opportunity for ROS-inducing agents to be employed as antitumor drugs. Moreover, the repair of 8-oxoG by OGG1-BER is an important event in cancer therapy strategies based on a ROS-mediated mechanism, so the use of a ROS-inducing agent, in combination with 8-oxoG repair enzyme inhibitors, may be an attractive new approach to improving therapeutic outcomes.

## Figures and Tables

**Table 1:** A series of inhibitors of BER process involved in Part 4.

Target	Inhibitor	Validation	Current Status *
MTH1	Karonudib (TH1579)	In vitro, Cell lines, Xenografts	Phase I
	TH588, TH287	In vitro, Cell lines, Xenografts	
	(S)-crizotinib	In vitro, Cell lines, Xenografts	
	IACS-4759, IACS-4619	In vitro, Cell lines	
APE1	TRC102 (Methoxyamine)	In vitro, Cell lines, Xenografts	Phase I/II
	Gossypol	In vitro, Cell lines, Xenografts	Phase III
	CRT0044876	In vitro, Cell lines	
	AR03	In vitro, Cell lines	
PARP1/2	Olaparib	In vitro, Cell lines, Xenografts	FDA-approved
	Talazoparib	In vitro, Cell lines, Xenografts	FDA-approved
	Niraparib	In vitro, Cell lines, Xenografts	FDA-approved
	Rucaparib	In vitro, Cell lines, Xenografts	FDA-approved
	Veliparib	In vitro, Cell lines, Xenografts	Phase III

* More details about inhibitors can be obtained from *ClinicalTrials.gov*. At present, there are many OGG1 inhibitors, such as TH5487 and O8. OGG1 inhibitors have a good effect on inhibiting inflammation, but they have no obvious effect on tumor therapy.
